# The Association of Psychological Factors With Willingness to Share Health-Related Data From Technological Devices: Cross-Sectional Questionnaire Study

**DOI:** 10.2196/64244

**Published:** 2025-01-23

**Authors:** Marijn Eversdijk, Emma Rixt Douma, Mirela Habibovic, Willem Johan Kop

**Affiliations:** 1 Department of Medical and Clinical Psychology, Center of Research on Psychological Disorders and Somatic Diseases (CoRPS), Tilburg University, Tilburg, the Netherlands, 31 134662142

**Keywords:** health data sharing, privacy concerns, wearable health technology, personality, psychological flexibility, optimism, social inhibition, psychological factors, willingness, health-related data, mobile phone

## Abstract

**Background:**

Health-related data from technological devices are increasingly obtained through smartphone apps and wearable devices. These data could enable physicians and other care providers to monitor patients outside the clinic or assist individuals in improving lifestyle factors. However, the use of health technology data might be hampered by the reluctance of patients to share personal health technology data because of the privacy sensitivity of this information.

**Objective:**

This study investigates to what extent psychological factors play a role in people’s willingness to share personal health technology data.

**Methods:**

Data for this cross-sectional study were obtained by quota sampling based on age and sex in a community-based sample (N=1013; mean age 48.6, SD 16.6 years; 522/1013, 51.5% women). Willingness to share personal health technology data and related privacy concerns were assessed using an 8-item questionnaire with good psychometric properties (Cronbach’s α=0.82). Psychological variables were assessed using validated questionnaires for optimism (Life Orientation Test—Revised), psychological flexibility (Psychological Flexibility Questionnaire), negative affectivity (Type D Scale-14—Negative Affectivity), social inhibition (Type D Scale-14—Social Inhibition), generalized anxiety (Generalized Anxiety Disorder-7), and depressive symptoms (Patient Health Questionnaire-9). Data were analyzed using multiple linear regression analyses, and network analysis was used to visualize the associations between the item scores.

**Results:**

Higher levels of optimism (β=.093; *P*=.004) and psychological flexibility (β=.127; *P*<.001) and lower levels of social inhibition (β=−.096; *P*=.002) were significantly associated with higher levels of willingness to share health technology data when adjusting for age, sex, and education level in separate regression models. Other associations with psychological variables were not statistically significant. Network analysis revealed that psychological flexibility clustered more with items that focused on the benefits of sharing data, while optimism was negatively associated with privacy concerns.

**Conclusions:**

The current results suggest that people with higher levels of optimism and psychological flexibility and those with lower social inhibition levels are more likely to share health technology data. The magnitude of the effect sizes was low, and future studies with additional psychological measures are needed to establish which factors identify people who are reluctant to share their data such that optimal use of devices in health care can be facilitated.

## Introduction

Recent advances in health technology, including wearable devices and smartphone apps, have facilitated remote patient health monitoring [1-3]. Sharing data from these devices with care providers, researchers, or device developers has multiple advantages for the patient, including early detection of health-related abnormalities, risk stratification, individualized health interventions, and overall better disease management [4,5]. The health care system might also benefit by applying machine learning algorithms to the large amounts of collected data to improve future health care and increase cost-efficiency [6].

Despite the potential health benefits, uptake of these health technologies is often impeded by patient and other user concerns related to the privacy and confidentiality of sharing sensitive personal data [7-9]. These privacy-related concerns are relevant because they influence the willingness to share their health technology data [10,11]. While respect for personal autonomy in the decision to share data is an important cornerstone in medical ethics, privacy-related objections could be overcome by adjusting the technological features of a device based on specific desires or patient characteristics [12]. Assessing individual differences in willingness to share technology data could help develop a personalized approach toward sharing health data to facilitate lower privacy concerns, higher user satisfaction, higher satisfaction with care, and better health outcomes.

Willingness to share personal health technology data can be defined as an individual tendency to allow others to have access to personal biobehavioral (eg, heart rate) or other personal data (eg, geographic location) collected using health technology (eg, wearable devices and smartphone apps) [11,13,14]. In general, the extent to which a person is willing to share or protect personal data is determined by an interaction between the sensitivity of the information (eg, medical or socioeconomic information, purchase history, and location), the context of data sharing (eg, health care or commercial setting), and personal dispositions such as privacy-related attitudes [15]. These personal dispositions are most relevant for the question of who is willing to share personal health technology data. Research has identified not only digital literacy, older age, and privacy knowledge as factors associated with willingness to share technology data but also psychological factors such as personality traits and generalized trust [13,16,17]. However, there is a knowledge gap regarding the magnitude of associations between psychological factors and willingness to share personal health technology data.

Current evidence regarding positive psychological factors, although scarce, indicates that patients who are overall more optimistic are more likely to expect positive outcomes from sharing their data (eg, better health monitoring) or are more likely to believe that they are less at risk of privacy breaches than others [18,19]. Optimism is also associated with higher psychological flexibility, which is the ability to distance from current mindsets and consider other possible mindsets [20,21]. People with high levels of psychological flexibility might be better able to act upon goals that involve new technologies to facilitate better personalized care that might also help other patients by sharing data even when this comes at the cost of less privacy for the user.

In addition to positive psychological traits, negative psychological factors are also expected to be associated with the willingness to share health-related data. Experiencing distress (eg, anxiety and depressive symptoms) is linked to threat-avoidant behaviors and reduced engagement in reward-seeking behaviors [22]. These tendencies might translate into overvaluing the risks and undervaluing the potential rewards of sharing data, leading to a lower willingness to share health technology data. Prior research has shown that anxiety and depressive symptoms are more likely to occur in patients with negative personality traits such as negative affectivity (NA) and social inhibition (SI) [23]. The relative importance of positive psychological characteristics versus negative characteristics with the willingness to share health technology data has yet to be researched.

This study explores associations between psychological factors and the willingness to share personal health technology data. It was expected that positive psychological characteristics (ie, optimism and psychological flexibility) would be associated with a higher willingness to share health technology data, whereas negative psychological characteristics (ie, anxiety, depressive symptoms, and measures of distress-related personality traits: NA and SI) would be associated with less willingness to share health technology data. The role of sociodemographic background factors (age, sex, and education level) and whether or not participants have previously used health technology apps was also investigated using multivariable models. Exploratory network analyses on the questionnaire items were conducted to reveal potential psychological mechanisms between the participant responses on the psychological constructs and items that reflect the willingness to share data.

## Methods

### Participants and Procedure

This cross-sectional study was based on data obtained from a Dutch community-based sample (N=1013) as part of an annually recurring survey study (for additional details, see the study by Kop et al [24]). Research assistants were instructed to apply quota sampling within their personal network by approaching a minimum number of participants from each sex and age group to ensure equal representation (eg, to ensure the number of men aged between 31 and 40 years in the total sample is equal to the number of women aged between 31 and 40 years in the total sample). Inclusion criteria were age above 18 years and being fluent in Dutch. Although there were no geographical restrictions, most participants lived in the south of the Netherlands, as a result of the sampling procedure. Data for this study were collected between February 2022 and April 2022. Data collection took place online using Qualtrics.

### Ethical Considerations

Ethical approval was obtained from the Tilburg School of Social and Behavioral Sciences ethical review board (protocol RP55). All participants gave informed consent before taking part in this study, and their data were deidentified prior to data analysis. Participants were not compensated for their participation.

### Measures

Data collection was conducted using a web-based survey after participants provided informed consent for their participation. All data were obtained using validated questionnaires or purpose-designed questions (see Multimedia Appendix 1 for an overview of the used questionnaires).

#### Health Technology Data-Sharing Inventory

The willingness to share health technology data was assessed using an 8-item questionnaire designed for the purposes of this study. Six questions focused on the potential benefits of sharing data (eg, “I would allow my personal data to be shared if it would help me”). Two items were derived from the Service User Technology Acceptability Questionnaire and focused on the feeling that health technology violates confidentiality or people’s privacy [25]. Participants were asked to indicate their level of agreement on a 5-point Likert scale (1=strongly disagree, 5=strongly agree). After reverse-coding the 2 negatively phrased items, a continuous total score was calculated by adding up the items (range 8-40). A higher score indicates a higher willingness to share health technology data. The Cronbach’s α coefficient for this scale was 0.82 in this study, which indicates a good internal consistency of this inventory.

#### Optimism

Optimism was measured using the 10-item version of the Life Orientation Test—Revised (LOT-R) [26]. The LOT-R includes 3 items about optimism, 3 items about pessimism, and 4 filler items. Examples of questions are “I’m always optimistic about my future” (ie, optimism) and “I hardly ever expect things to go my way” (ie, pessimism). Participants were asked to indicate their level of agreement on a 5-point Likert scale (0=strongly disagree, 4=strongly agree). A continuous total score for optimism (range 0‐24) was calculated by adding the scores on optimism to the reversed scores on pessimism. A higher score indicates higher levels of optimism. The published internal consistency of the LOT-R total score is acceptable (Cronbach’s α=0.78) [26], which was similar and acceptable in the present sample (Cronbach’s α=0.74).

#### Psychological Flexibility

The Psychological Flexibility Questionnaire (PFQ) [27] was used to measure psychological flexibility. Participants were asked to indicate their degree of agreement on a 5-point Likert scale (1=strongly disagree, 5=strongly agree), enabling participants to enter a neutral response (3=neither agree nor disagree). Examples of items are “I feel open to changes” and “Concepts may possess different meanings when perceived in different contexts.” The answers to all 20 questions were added up to a continuous total score (range 20-100). A higher score indicates higher psychological flexibility. Internal consistency of the questionnaire was good in the present sample (Cronbach’s α=0.89).

#### Negative Affectivity and Social Inhibition

NA and SI were measured using the DS-14 (Type D Scale-14) [28]. These scales have been developed to assess a construct identifying individuals with a distressed personality trait (ie, type D), but the subscales are used as separate dimensions in this paper, as the focus was not on the interaction of these 2 subscales, which is needed when investigating the type D construct [29]. The questionnaire contains a 7-item subscale assessing NA and a 7-item scale assessing SI. An example of a question for NA is “I often feel unhappy,” and for SI, an example is “I am a closed kind of person.” Two questions of the SI subscale are reverse-phrased and recoded before calculating total scores. Participants rated their agreement on a 5-point Likert scale (0=strongly disagree, 4=strongly agree). A continuous total score was calculated by adding the scores on each subscale (range 0‐28). A higher score indicates a higher presence of the personality trait. The Cronbach’s α value for NA in this sample was 0.88 and for SI 0.88, indicating good internal consistency.

#### Generalized Anxiety and Depressive Symptoms

The Generalized Anxiety Disorder-7 (GAD-7) questionnaire [30] was used to measure the presence of anxiety-related complaints over the last 2 weeks (example items are “Feeling nervous, anxious or on edge” and “Not being able to stop or control worrying”). Participants were asked to indicate the presence of these complaints on a 4-point Likert scale (0=not at all, 3=nearly every day). A continuous total score was calculated by adding the scores of the 7 items (range 0‐21). A higher score indicates a higher presence of anxiety-related complaints. The Cronbach’s α value in this study was 0.87, indicating good internal consistency.

Depressive symptoms were assessed using the Patient Health Questionnaire-9 (PHQ-9) [31], asking about the presence of depressive symptoms such as “Little interest or pleasure in doing things” or “Feeling down, depressed, or hopeless.” Questions focused on the presence of complaints over the past 2 weeks and had to be filled in on a 4-point Likert scale (0=not at all, 3=nearly every day). A continuous total score was calculated by adding the scores on all 9 items (range 0-27). A higher score indicates a higher presence of depressive symptoms. Internal consistency was considered good, with a Cronbach’s α value of 0.85 in the present sample.

#### Background Variables and Covariates

Demographic measures (age, self-reported sex, and education level) were obtained from the self-report questionnaire. The dataset contained only a measurement for sex and not for gender. Although gender would have better matched the psychological and behavioral variables in the dataset, the authors decided to report sex since this measure better represents the construct measured in the questionnaire. Level of education was divided into low (completed secondary school or less), middle (completed vocational education or high school only), or high (completed college or higher). Participants also reported their health status by indicating the presence of a lifetime diagnosed medical condition (eg, cardiovascular, cancer, lung disease, gastrointestinal disorder, and diabetes mellitus). In addition, an assessment was made concerning participants’ use of smartphone-based health apps or related wearable technology.

### Statistical Analysis

Descriptive statistics were obtained for all demographic and psychological variables by calculating the mean and SD for continuous variables and the number of occurrences and percentages for all categorical variables. Pearson and nonparametric Spearman correlation analyses were used to obtain an overview of the bivariate associations between the psychological measures and willingness to share health technology data. For illustrative purposes, odds ratios (ORs) with 95% CIs were also calculated using tertile-based dichotomized data of the psychological measures and the willingness to share health data (a value higher than 1 indicates a higher chance of willingness to share data based on the upper tertile of the psychological measure).

Multiple linear regression analyses were performed to assess which variables were independently associated with the willingness to share health technology data. Assumptions for regression analysis, including linearity, normality of residuals, homoscedasticity, and the presence of influential cases, were evaluated using the plot function in R (version 4.2.2; R Foundation for Statistical Computing) (findings are shown in Table S1 and Figure S1 in Multimedia Appendix 2). Data are presented in terms of the overall explained variance of the model (*R*^2^) and the standardized regression weights per variable (β). Analyses first focused on the results of the full sample (N=1013). Subsequent sensitivity analyses were conducted for participants who had used or currently used health technology apps (N=244) and those not using such apps (N=769).

Total scores were coded as missing values if participants answered <60% of all items of that specific scale. If at least 60% of the items of a questionnaire were completed, then missing items of that scale were imputed by the mean of the answered questions of that scale. The percentage of missing values among all the psychological item scores was <0.1%, and there appeared to be no patterns in missingness. For the multiple regression models, listwise deletion was used to deal with missing values on the total scores of each predictor. The potential of biased parameters estimated as a result of mean imputation was limited, as it was applied only within variables, the percentage of missing values was extremely low, and listwise deletion was used [32].

Exploratory network analyses were performed to further investigate the associations between the item scores of all the psychological scales. Network analyses were visualized with the “qgraph” package (version 1.9.3) in R [33]. Each node represents an individual item of the questionnaire, while edges represent the associations between 2 nodes (ie, questionnaire items).

## Results

### Participants and Demographics

Table 1 displays the participant characteristics. The mean age was 48.6 (SD 16.6) years, and 51.5% (522/1013) of the participants were women. Levels of education were relatively high (52.3% higher education). A current or history of a medical disorder was reported by 36.9% (374/1013) of the participants, and average anxiety and depressive symptom levels were relatively low.

**Table 1.** Characteristics of all included participants.

	Total sample (N=1013)	Technology users (N=244)	Not using health technology (N=769)	*P* value
Age (years), mean (SD)	48.6 (16.6)	46.3 (15.0)	49.4 (17.1)	.01
Sex (female), n (%)	522 (51.5)	127 (52.0)	395 (51.4)	.91
Education, n (%)				<.001
Low	129 (12.7)	26 (10.7)	103 (13.4)	
Middle	354 (34.9)	65 (26.6)	289 (37.6)	
High	530 (52.3)	153 (62.7)	377 (49.0)	
Lifetime medical disorder, n (%)	374 (36.9)	94 (38.5)	280 (36.4)	.60
Variables (range), mean (SD)				
Optimism (0-24)	15.5 (3.5)	16.0 (3.5)	15.4 (3.4)	.02
Psychological flexibility (20-100)	75.1 (8.6)	76.6 (9.1)	74.6 (8.4)	.002
Negative affectivity (0-28)	7.7 (5.2)	7.2 (4.8)	7.9 (5.4)	.09
Social inhibition (0-28)	8.7 (5.3)	8.7 (5.1)	8.7 (5.4)	.87
Generalized anxiety (0-21)	3.0 (3.4)	3.0 (3.3)	2.9 (3.4)	.77
Depressive symptoms (0-27)	3.5 (3.9)	3.4 (3.2)	3.6 (4.1)	.42
Health data sharing (8-40)	23.8 (6.0)	25.0 (5.6)	23.4 (6.0)	<.001

The variable willingness to share health technology was approximately normally distributed, with scores ranging from 8 to 40 (mean 23.8, SD 6.0, median 24.0) (Figure S2 in Multimedia Appendix 2). Furthermore, 24.1% (244/1013) of the participants were currently using digital health apps or other forms of health technology (ie, wearable devices or smartphone apps aimed at improving physical activity, sleep, and medication adherence, or at reducing stress, alcohol use, and smoking). Participants who used technological devices were younger, were more likely to have a high education level, and had higher psychological flexibility levels than participants who did not use such devices, whereas no differences were found on the other variables listed in Table 1.

### Associations of Psychological Factors With Willingness to Share Health Technology Data

As shown in Table 2, high levels of optimism (*r*=0.07; *P*=.03) and psychological flexibility (*r*=0.13; *P*<.001) were significantly correlated with higher levels of willingness to share health technology data. In addition, higher levels of SI were associated with a lower willingness to share health technology data (*r*=−0.08; *P*=.01). Other bivariate correlations between psychological factors and willingness to share data were nonsignificant. When repeating these analyses with dichotomized scores, it was found that participants scoring in the highest tertile of psychological flexibility were 63% more likely to score high (upper tertile) on willingness to share health technology data (OR 1.63, 95% CI 1.25-2.12). Participants scoring in the lowest tertile of SI were also more likely to be willing to share their data (OR 1.53, 95% CI 1.17-2.00), whereas none of the other dichotomized psychological measures revealed significant ORs with a high level of willingness to share data.

**Table 2.** Correlation and regression coefficients of a linear regression model with all psychological measures and willingness to share health technology data.

Variable	Spearman ρ	*P* value	Pearson *r*	*P* value	*B* (95% CI)	β	*P* value
Optimism (LOT-R^a^)	0.07	.03	0.07	.03	0.094 (−0.043 to 0.231)	.054	.18
Psychological flexibility (PFQ^b^)	0.13	<.001	0.13	<.001	0.067 (0.017 to 0.117)	.097	.008
Negative affectivity (DS-14: NA^c^)	−0.04	.20	−0.04	.26	0.050 (−0.067 to 0.166)	.043	.41
Social inhibition (DS-14: SI^d^)	−0.10	.002	−0.08	.01	−0.068 (−0.149 to 0.013)	−.061	.10
Generalized anxiety (GAD-7^e^)	0.01	.80	0.01	.84	0.115 (−0.072 to 0.301)	.065	.23
Depressive symptoms (PHQ-9^f^)	−0.01	.71	−0.02	.62	−0.097 (−0.257 to 0.063)	−.063	.23
Age	−0.08	.01	−0.09	.004	−0.031 (−0.055 to −0.007)	−.086	.01
Sex^g^	0.07	.04	0.07	.03	0.873 (0.124 to 1.622)	.073	.02
Education level^h^	−0.06	.05	−0.06	.06	−0.629 (−1.160 to −0.096)	−.074	.02

a LOT-R: Life Orientation Test—Revised.

b PFQ: Psychological Flexibility Questionnaire.

c DS-14 NA: Type D Scale-14 Negative Affectivity

d DS-14 SI: Type D Scale-14 Social Inhibition

e GAD-7: Generalized Anxiety Disorder-7.

f PHQ-9: Patient Health Questionnaire-9.

g Sex coded as 0 (female) and 1 (male; a positive value indicates that men have higher willingness to share health technology data than women).

h Education level coded as 0 (low), 1 (middle), and 2 (high; a positive value indicates that a higher education is associated with a higher willingness to share health technology data).

Of the background factors, male participants (Cohen *d*=−0.14; *P*=.03) and technology users (Cohen *d*=−0.25; *P*<.001) scored significantly higher on willingness to share technology data (Table S2 in Multimedia Appendix 2). When adjusting for age, sex, and education level in separate linear regression models, optimism (β=.093; *P*=.004), psychological flexibility (β=.127; *P*<.001), and SI (β=−.096; *P*=.002) remained significantly associated with willingness to share health technology data.

To evaluate which psychological factor was associated with willingness to share health technology data, independent of other psychological factors, a multiple linear regression analysis was used entering all psychological factors simultaneously while also adjusting for age, sex, and education level (adjusted *R*^2^=0.03; *P*<.001). Higher psychological flexibility was the psychological variable that was independently associated with more willingness to share data (β=.097; *P*=.008) while adjusting for covariates. Other correlates of willingness to participate in health technology data sharing were younger age (β=−.086; *P*=.01), male sex (β=.073; *P*=.02), and lower education level (β=−.074; *P*=.02) (Table 2).

When repeating the analyses after stratifying the sample by the use of health technology, a similar pattern of results was found for both groups. Among health technology users (N=244), higher levels of psychological flexibility were independently associated with more willingness to share data (β=.204; *P*=.006). Other associations with the willingness to share data among technology users were nonsignificant (adjusted *R*^2^ for the full model=0.04; *P*=.03). In the subset of participants who did not use health technology (N=769), lower SI was associated with a higher willingness to share data (β=−.092; *P*=.03), while male sex (β=.076; *P*=.04) and lower education level (β=−.089; *P*=.02) were also independently associated with willingness to share data (Table S3 in Multimedia Appendix 2).

### Network Analysis of the Interrelationship Between Psychological Factors and Willingness to Share Health Technology Data

**Figure 1.**
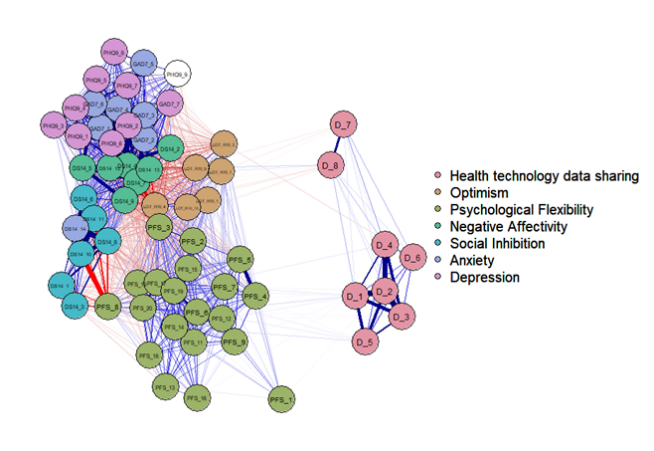
Network correlation structure of all psychological measurements and willingness to share health technology data.

Figure 1 shows the results of the network analysis based on the correlational structure of all questionnaire items. Every edge represents a correlation larger than 0.1. Each underlying construct is represented by a different color. The network visualizes the extent to which items cluster together, indicating the measurement of the same underlying construct. The network visualizes that the items on data sharing do not cluster together with most of the psychological predictors. Within the health technology data-sharing items, it appeared that items 7 (“I am worried about the confidentiality of the private information being exchanged by the health technology”) and 8 (“Health technology will violate my privacy”) were distinct from the other items (eg, “I would allow my personal data to be shared with my physician if it would improve my treatment”), based on their relative spatial position. The item-level comparisons show that items 7 and 8 cluster more toward items on optimism, while the other data-sharing items cluster more toward psychological flexibility. A regularized network analysis can be found in Figure S3 in Multimedia Appendix 2.

## Discussion

### Principal Findings

This study shows that higher levels of optimism and psychological flexibility and lower levels of SI are associated with higher levels of willingness to share health technology data. Multivariable analyses further indicated that psychological flexibility was the strongest and independent psychological factor associated with the willingness to share health technology data. Younger age, male sex, and lower education level were also independently associated with a higher willingness to share health technology data. Item-level network analysis revealed that optimism was associated with less concerns about confidentiality and privacy, whereas psychological flexibility was associated with a higher willingness to share health technology data if it could benefit the user or others.

### Integration of the Present Findings With the Existing Literature

The current findings are consistent with previous research indicating that positive psychological traits, such as optimism and trust, are associated with a higher willingness to share health data [17,19]. The association of psychological flexibility with willingness to share health technology data has not yet been described in the scientific literature. People high on psychological flexibility are able to adapt to situational demands and balance competing desires, needs, and life domains [20]. Furthermore, they are more open toward new forms of behavior or shifting behavioral repertoires to achieve goals related to their personal values, which could play a central role in the decision to share personal health technology data with others. When balancing between giving up a part of their privacy compared and helping themselves or others by sharing their data, their prosocial values can take precedence over their protective intuitions, resulting into a higher willingness to share data. By shifting behaviors toward sharing data, people accept potential privacy concerns and remain true to personal values such as helping others, pursuing optimal health outcomes, or other values related to the benefits of data sharing [34].

### Limitations and Strengths

The findings of this study need to be interpreted in the context of several limitations. The questionnaire to evaluate willingness to share health technology data has not been validated in prior settings, although the psychometric properties of the questionnaire were good. There was a lack of diversity in the sample with regard to education level and ethnic background. The levels of generalized anxiety and depressive symptoms were also low in this community-based sample, potentially resulting in a floor effect for their relationship with willingness to share health data. The cross-sectional design precludes causal inferences and a more in-depth qualitative research approach, such as conducting individual interviews or focus groups, might provide additional information about the underlying values and arguments in the decision to share health technology data. However, even with additional knowledge on these motivations, there is a gap between data-sharing intention and action [35]. This observation indicates the need for future studies measuring actual privacy-related behaviors instead of evaluating intentions to engage in such behaviors. A strength of this study is the large sample covering a wide range of ages and an equal number of women and men, in which the relative importance of multiple psychological constructs could be studied. However, the set of psychological constructs was limited in the already existing dataset, and the models indicated very small explained variance, which pleads for an extension of other relevant psychological factors, such as technological self-efficacy, performance expectancy, trust, or other personality traits [14,17,36-39].

### Conclusions and Future Directions

This study documents a significant association of optimism, psychological flexibility, and low SI with a higher likelihood of individuals being willing to share health technology data. Of these variables, psychological flexibility appeared to be the strongest factor, particularly among individuals who use health technology. In addition, younger age, male sex, and low education levels were associated with a higher willingness to share health technology data, particularly among people who currently do not use health technology. Examining the underlying network structure of a large set of psychological variables revealed that psychological flexibility is mainly associated with the benefits of sharing health data, while people low on optimism show more concerns about confidentiality and privacy. Still, the magnitude of effect sizes was low, and additional work is needed on the underlying psychological mechanisms and reasons behind the disclosure and sharing of health-related data in general and health technology data in particular. Future studies that investigate psychological factors with reasons for sharing health data could identify which specific reasons are important for whom on an individual level instead of a population level. Additional studies with a broader set of psychological variables should be conducted to identify those variables that contribute the most to the willingness to share health data before specific clinical implications can be formulated. To increase the explained variance of the regression models, a broader set of psychological measures should be considered in studying their relationship with the willingness to share health technology data. Findings from these studies would provide important building blocks for the design of future health technology and promote individually tailored functionalities. This would enable customization of the data-sharing settings of personal health technology data sharing in accordance with individual preferences, rather than a binary decision to share all data or no data at all. The possibility to retract or modify prior consent to health technology data sharing with minimal effort could also increase people’s initial participation in data sharing [40]. This personalized approach might result in less privacy concerns, higher user satisfaction with the technology, higher satisfaction with health care, and potentially also better health outcomes.

## Supplementary material

10.2196/64244 Multimedia Appendix 1 Overview of questionnaire items used for analysis.

10.2196/64244 Multimedia Appendix 2 Supplemental tables and figures with further data on continuous psychological variables, dichotomous background factors, willingness to share data, and the regularized correlation structure of all scales.

## Abbreviations

DS-14: Type D Scale-14
GAD-7: Generalized Anxiety Disorder-7
LOT-R: Life Orientation Test—Revised
NA: negative affectivity
OR: odds ratio
PFQ: Psychological Flexibility Questionnaire
PHQ-9: Patient Health Questionnaire-9
SI: social inhibition

## References

[1] Afshin A, Babalola D, Mclean M (2016). Information technology and lifestyle: a systematic evaluation of internet and mobile interventions for improving diet, physical activity, obesity, tobacco, and alcohol use. JAHA.

[2] Chaudhry B, Wang J, Wu S (2006). Systematic review: impact of health information technology on quality, efficiency, and costs of medical care. Ann Intern Med.

[3] Habibović M, Rollman B (2023). Technological innovations in biobehavioral and psychosomatic medicine. Psychosom Med.

[4] Banerjee S, Hemphill T, Longstreet P (2018). Wearable devices and healthcare: data sharing and privacy. Inf Soc.

[5] Bayoumy K, Gaber M, Elshafeey A (2021). Smart wearable devices in cardiovascular care: where we are and how to move forward. Nat Rev Cardiol.

[6] Bauer M, Glenn T, Monteith S, Bauer R, Whybrow PC, Geddes J (2017). Ethical perspectives on recommending digital technology for patients with mental illness. Int J Bipolar Disord.

[7] Martinez-Martin N, Luo Z, Kaushal A (2021). Ethical issues in using ambient intelligence in health-care settings. Lancet Digit Health.

[8] Segura Anaya LH, Alsadoon A, Costadopoulos N, Prasad PWC (2018). Ethical implications of user perceptions of wearable devices. Sci Eng Ethics.

[9] Baines R, Stevens S, Austin D (2024). Patient and public willingness to share personal health data for third-party or secondary uses: systematic review. J Med Internet Res.

[10] Wetzels M, Broers E, Peters P, Feijs L, Widdershoven J, Habibovic M (2018). Patient perspectives on health data privacy and management: “Where Is my data and whose is it?”. Int J Telemed Appl.

[11] Kim TK, Choi M (2019). Older adults’ willingness to share their personal and health information when adopting healthcare technology and services. Int J Med Inform.

[12] Rubeis G, Schochow M, Steger F (2018). Patient autonomy and quality of care in telehealthcare. Sci Eng Ethics.

[13] Karampela M, Ouhbi S, Isomursu M (2019). Connected health user willingness to share personal health data: questionnaire study. J Med Internet Res.

[14] Rising CJ, Gaysynsky A, Blake KD, Jensen RE, Oh A (2021). Willingness to share data from wearable health and activity trackers: analysis of the 2019 Health Information National Trends Survey Data. JMIR Mhealth Uhealth.

[15] Brough AR, Martin KD (2020). Critical roles of knowledge and motivation in privacy research. Curr Opin Psychol.

[16] Baruh L, Secinti E, Cemalcilar Z (2017). Online privacy concerns and privacy management: a meta-analytical review. J Commun.

[17] Bansal G, Zahedi FM, Gefen D (2016). Do context and personality matter? Trust and privacy concerns in disclosing private information online. Inf Manag.

[18] Kokolakis S (2017). Privacy attitudes and privacy behaviour: a review of current research on the privacy paradox phenomenon. Comput Secur.

[19] Cho H, Lee JS, Chung S (2010). Optimistic bias about online privacy risks: testing the moderating effects of perceived controllability and prior experience. Comput Human Behav.

[20] Kashdan TB, Rottenberg J (2010). Psychological flexibility as a fundamental aspect of health. Clin Psychol Rev.

[21] Demirtaş AS (2020). Optimism and happiness in undergraduate students: cognitive flexibility and adjustment to university life as mediators. Anales de Psicología.

[22] Bishop SJ, Gagne C (2018). Anxiety, depression, and decision making: a computational perspective. Annu Rev Neurosci.

[23] Mols F, Denollet J (2010). Type D personality among noncardiovascular patient populations: a systematic review. Gen Hosp Psychiatry.

[24] Kop WJ, Toussaint A, Mols F, Löwe B (2019). Somatic symptom disorder in the general population: associations with medical status and health care utilization using the SSD-12. Gen Hosp Psychiatry.

[25] Hirani SP, Rixon L, Beynon M (2017). Quantifying beliefs regarding telehealth: Development of the Whole Systems Demonstrator Service User Technology Acceptability Questionnaire. J Telemed Telecare.

[26] Scheier MF, Carver CS, Bridges MW (1994). Distinguishing optimism from neuroticism (and trait anxiety, self-mastery, and self-esteem): a reevaluation of the Life Orientation Test. J Pers Soc Psychol.

[27] Ben-Itzhak S, Bluvstein I, Maor M (2014). The Psychological Flexibility Questionnaire (PFQ): development, reliability and validity. WebmedCentral Psychology.

[28] Denollet J (2005). DS14: standard assessment of negative affectivity, social inhibition, and type D personality. Psychosom Med.

[29] Lodder P (2020). Modeling synergy: how to assess a Type D personality effect. J Psychosom Res.

[30] Spitzer RL, Kroenke K, Williams JBW, Löwe B (2006). A brief measure for assessing generalized anxiety disorder: the GAD-7. Arch Intern Med.

[31] Kroenke K, Spitzer RL, Williams JB (2001). The PHQ-9: validity of a brief depression severity measure. J Gen Intern Med.

[32] Lodder P, Mellenbergh GJ, Adler HJ (2014). Advising on Research Methods: Selected Topics 2013.

[33] Epskamp S, Cramer AOJ, Waldorp LJ, Schmittmann VD, Borsboom D (2012). qgraph: network visualizations of relationships in psychometric data. J Stat Softw.

[34] Kalkman S, van Delden J, Banerjee A, Tyl B, Mostert M, van Thiel G (2022). Patients’ and public views and attitudes towards the sharing of health data for research: a narrative review of the empirical evidence. J Med Ethics.

[35] Kim J, Im E, Kim H (2023). From intention to action: the factors affecting health data sharing intention and action. Int J Med Inform.

[36] Chandrasekaran R, Katthula V, Moustakas E (2021). Too old for technology? Use of wearable healthcare devices by older adults and their willingness to share health data with providers. Health Informatics J.

[37] Tolentino DA, Costa DK, Jiang Y (2023). Determinants of American adults’ use of digital health and willingness to share health data to providers, family, and social media: a cross-sectional study. Comput Inform Nurs.

[38] Cherif E, Bezaz N, Mzoughi M (2021). Do personal health concerns and trust in healthcare providers mitigate privacy concerns? Effects on patients’ intention to share personal health data on electronic health records. Soc Sci Med.

[39] Bansal G, Zahedi FM, Gefen D (2010). The impact of personal dispositions on information sensitivity, privacy concern and trust in disclosing health information online. Decis Support Syst.

[40] Budin-Ljøsne I, Teare HJA, Kaye J (2017). Dynamic consent: a potential solution to some of the challenges of modern biomedical research. BMC Med Ethics.

