# Supplementary material for: The Association of Psychological Factors With Willingness to Share Health-Related Data From Technological Devices: Cross-Sectional Questionnaire Study
Source: JMIR Form Res. 2025 Jan 23;9:e64244. doi: 10.2196/64244 (PMC11780973; doi:10.2196/64244)
Supplement: Multimedia Appendix 1 [file formative-v9-e64244-s001.docx]

**Multimedia Appendix 1**

Overview of questionnaire items used for analysis (translated from Dutch)

Sociodemographic variables (age, sex, education level, medical condition, technology use)

1. Please indicate your age
2. What was your sex at birth? (Selected choice: male/female/other)
3. What is your highest completed level of education? (Selected choice: Don’t know/ Preschool / Primary education / Secondary education / High school / Vocational school / Associate degree / Bachelor degree / Master Degree / Doctoral Degree)
4. Has one of the following conditions been diagnosed by a physician currently or in the past? Multiple answers allowed. (Cardiovascular disease/ Asthma/ Lung condition/ Cancer/ Diabetes Mellitus/ Gastrointestinal disorder/ Hypertension/ Depression/ Anxiety disorder/ Other, namely …)
5. Do you currently use any form of technology to monitor or improve your health aimed at changing your behaviour? Multiple answers allowed. (Physical activity tracker (e.g. FitBit, Garmin smartwatch, Oura ring) / Sleeptracker (e.g. FitBit, Garmin, Oura) / Smartphone app for monitoring or improving nutrition / Smartphone app for monitoring or improving sleep / Smartphone app for monitoring or improving medication adherence / Smartphone app for monitoring or improving stress levels / Smartphone app for monitoring or improving physical activity / Smartphone app for monitoring or improving smoking behaviour / Smartphone app for monitoring or improving alcohol consumption / Other, namely …)

Health technology data sharing (1 = strongly disagree, 2 = disagree, 3 = neither agree nor disagree, 4 = agree, 5 = strongly agree)

1. My personal data may be used to better help me.
2. My personal data may be used to better help other people using health technology.
3. My personal data may be used to develop new health technology, as long as the technology company does not make a profit from using my data.
4. My personal data may be used to develop new health technology, and as far as I am concerned, the technology company may also benefit (make a profit) from the use of my data.
5. My data may be forwarded to my doctor, so that my treatment can be improved.
6. My data may be forwarded to my health insurer so that my premium or the premium of other people can be adjusted.
7. I am concerned about the confidentiality of the private information that is exchanged by health technology.^1^
8. Health technology could violate my privacy.^1^

1. Modified from the Service User Technology Acceptability Questionnaire (SUTAQ) [25]

Revised Life Orientation Test (LOT-R) [26]. Please answer the following questions about yourself by indicating the extent of your agreement using the following scale: 0 = strongly disagree, 1 = disagree, 2 = neutral, 3 = agree, 4 = strongly agree.

1. In uncertain times, I usually expect the best
2. It’s easy for me to relax (filler)
3. If something can go wrong for me, it will (-)
4. I’m always optimistic about my future
5. I enjoy my friends a lot (filler)
6. It’s important for me to keep busy (filler)
7. I hardly ever expect things to go my way (-)
8. I don’t get upset too easily (filler)
9. I rarely count on good things happening to me (-)
10. Overall, I expect more good things to happen to me than bad

Psychological flexibility (PFQ) [27] (1 = strongly disagree, 2 = disagree, 3 = neither agree nor disagree, 4 = agree, 5 = strongly agree)

1. Reality is never absolute
2. In situations of changeable reality I am able to initiate the required changes
3. When times are hard, even very hard, I am able to remember that there are better times ahead
4. Concepts may possess different meanings when perceived in different contexts
5. There are usually many possible ways to do things
6. I am open to experiencing the different and the exceptional
7. In a disagreement there are always numerous possible solutions – you just have to find them
8. I am an open person in comparison with others
9. Reality has many different aspects
10. Often find a change to be a challenge
11. I think of myself as a person who is attentive to a variety of different messages and ideas
12. I find it easy to acknowledge reality’s multiversity of manifestations, manifestations that may often be significantly divergent, or even conflict with one another
13. It is important to me to learn from each and every person
14. I recognize myself as someone who is able to change his/her position and modify him/herself accordingly
15. I feel ready to accept future changes
16. At times I can make significant decisions, based on my need to change
17. When I encounter difficulties in achieving a goal, I am able to try numerous different solutions
18. It is easy for me to think of ways of conduct that are very unconventional
19. I feel open to changes
20. When giving an assignment I am able to come up with original ways of completing it, in comparison with how I used to approach it before

Negative affectivity (NA) and social inhibition (SI) [28] (1 = strongly disagree, 2 = disagree, 3 = neither agree nor disagree, 4 = agree, 5 = strongly agree)

1. I make contact easily when I meet people (-) (SI)
2. I often make a fuss about unimportant things (NA)
3. I often talk to strangers (-) (SI)
4. I often feel unhappy (NA)
5. I am often irritated (NA)
6. I often feel inhibited in social interactions (SI)
7. I take a gloomy view of things (NA)
8. I find it hard to start a conversation (SI)
9. I am often in a bad mood (NA)
10. I am a closed kind of person (SI)
11. I would rather keep other people at a distance (SI)
12. I often find myself worrying about something (NA)
13. I am often down in the dumps (NA)
14. When socializing, I don’t find the right things to talk about (SI)

Patient Health Questionnaire (PHQ-9) [31]. Over the last *2 weeks*, how often have you been bothered by any of the following problems? (0 = not at all, 1= several days, 2 = more than half the days, 3 = nearly every day)

1. Little interest or pleasure in doing things
2. Feeling down, depressed, or hopeless
3. Trouble falling or staying asleep, or sleeping too much
4. Feeling tired or having little energy
5. Poor appetite or overeating
6. Feeling bad about yourself – or that you are a failure or have let yourself or your family down
7. Trouble concentrating on things, such as reading the newspaper or watching television
8. Moving or speaking so slowly that other people could have noticed. Or the opposite – being so fidgety or restless that you have been moving around a lot more than usual
9. Thoughts that you would be better off dead, or of hurting yourself

Generalised Anxiety Disorder (GAD-7) Questionnaire [30]. Over the last *two weeks,* how often have you been bothered by the following problems? (0 = not at all, 1 = several days, 2 = more than half of the days, 3 = nearly every day)

1. Feeling nervous, anxious, or on edge
2. Not being able to stop or control worrying
3. Worrying too much about different things
4. Trouble relaxing
5. Being so restless that it is hard to sit still
6. Becoming easily annoyed or irritable
7. Feeling afraid, as if something awful might happen
