# Supplementary material for: The Association of Psychological Factors With Willingness to Share Health-Related Data From Technological Devices: Cross-Sectional Questionnaire Study
Source: JMIR Form Res. 2025 Jan 23;9:e64244. doi: 10.2196/64244 (PMC11780973; doi:10.2196/64244)
Supplement: Multimedia Appendix 2 [file formative-v9-e64244-s002.docx]

**Multimedia Appendix 2**

**Supplemental tables and figures**

**Table S1**

*Pearson correlation coefficients of all continuous psychological variables and age*

|  | 1. | 2. | 3. | 4. | 5. | 6. | 7. | 8. |
| --- | --- | --- | --- | --- | --- | --- | --- | --- |
| 1. Health technology data sharing | - |  |  |  |  |  |  |  |
| 2. Optimism | .07* | - |  |  |  |  |  |  |
| 3. Psychological flexibility | .13** | .44** | - |  |  |  |  |  |
| 4. Negative affectivity | -.04 | -.58** | -.35** | - |  |  |  |  |
| 5. Social inhibition | -.08* | -.34** | -.37** | .47** | - |  |  |  |
| 6. Generalised anxiety | .01 | -.42** | -.19** | .69** | .30** | - |  |  |
| 7. Depressive symptoms | -.02 | -.41** | -.16** | .67** | .32** | .78** | - |  |
| 8. Age | -.09* | .14** | -.03 | -.22** | -.11** | -.32** | -.34** | - |

* *p* < .05. ** *p* < .001.

*Spearman correlation coefficients of all continuous psychological variables and age*

|  | 1. | 2. | 3. | 4. | 5. | 6. | 7. | 8. |
| --- | --- | --- | --- | --- | --- | --- | --- | --- |
| 1. Health technology data sharing | - |  |  |  |  |  |  |  |
| 2. Optimism | .07* | - |  |  |  |  |  |  |
| 3. Psychological flexibility | .13** | .43** | - |  |  |  |  |  |
| 4. Negative affectivity | -.04 | -.53** | -.35** | - |  |  |  |  |
| 5. Social inhibition | -.10* | -.32** | -.37** | .47** | - |  |  |  |
| 6. Generalised anxiety | .01 | -.36** | -.17** | .66** | .27** | - |  |  |
| 7. Depressive symptoms | -.01 | -.32** | -.12** | .60** | .28** | .73** | - |  |
| 8. Age | -.08* | .10* | -.06 | -.19** | -.08* | -.32** | -.35** | - |

* *p* < .05. ** *p* < .001.

**Table S2**

*Association between dichotomous background factors with willingness to share data*

|  |  | Mean (SD) | *t*- or *F*- value | *p*-value |
| --- | --- | --- | --- | --- |
| Sex | Female | 23.4 (5.7) |  |  |
|  | Male | 24.2 (6.2) | -2.13 | .033 |
| Education | Low | 24.5 (5.4) |  |  |
|  | Middle | 24.0 (5.7) |  |  |
|  | High | 23.5 (6.3) | 1.82 | .163 |
| Medical or mental disorder | Yes | 23.9 (5.9) |  |  |
|  | No | 23.7 (6.0) | -0.46 | .642 |
| Health technology use | Yes | 25.0 (5.6) |  |  |
|  | No | 23.4 (6.1) | 3.51 | <.001 |

**Table S3**

*Multiple regression coefficients for the association of psychological factors with willingness to share health technology data*

|  | Total sample |  | Technology users |  | Not using health technology |  |
| --- | --- | --- | --- | --- | --- | --- |
|  | *b* [95% CI] | *β* | *b* [95% CI] | *β* | *b* [95% CI] | *β* |
| LOT_R | 0.094 [-0.043, 0.231] | 0.054 | 0.137 [-0.119, 0.393] | 0.086 | 0.084 [-0.078, 0.246] | 0.048 |
| PFQ | 0.067 [0.017, 0.117] | 0.097* | 0.125 [0.036, 0.214] | 0.204* | 0.037 [-0.023, 0.096] | 0.051 |
| NA | 0.050 [-0.067, 0.166] | 0.043 | 0.077 [-0.182, 0.336] | 0.065 | 0.059 [-0.074, 0.192] | 0.052 |
| SI | -0.068 [-0.149, 0.013] | -0.061 | -0.001 [-0.153, 0.152] | -0.001 | -0.103 [-0.199, -0.008] | -0.092* |
| GAD-7 | 0.115 [-0.072, 0.301] | 0.065 | -0.193 [-0.568, 0.183] | -0.113 | 0.167 [-0.050, 0.383] | 0.094 |
| PHQ-9 | -0.097 [-0.257, 0.063] | -0.063 | 0.171 [-0.226, 0.567] | -0.096 | -0.135 [-0.312, 0.042] | -0.092 |
| Age | -0.031 [-0.055, -0.007] | -0.086* | -0.048 [-0.102, 0.005] | -0.129 | -0.024 [-0.051, 0.003] | -0.068 |
| Sex ^a^ | 0.873 [0.124, 1.622] | 0.073* | 0.934 [-0.591, 2.459] | 0.083 | 0.920 [0.048, 1.791] | 0.076* |
| Edu | -0.629 [-1.160, -0.096] | -0.074* | -0.670 [-1.772, 0.431] | -0.081 | -0.760 [-1.373, -0.148] | -0.089* |

Abbreviations: LOT_R: Life Orientation Test Revised; PFQ: Psychological Flexibility Questionnaire; NA: Negative Affectivity; SI: Social Inhibition; GAD-7: Generalized Anxiety Disorder-7; PHQ-9: Patient Health Questionnaire-9; Edu: Education level.

^a^ Sex coded as 0 (female) and 1 (male; a positive value indicates that men have higher willingness to share health technology data than women).

* *p* < .05

**
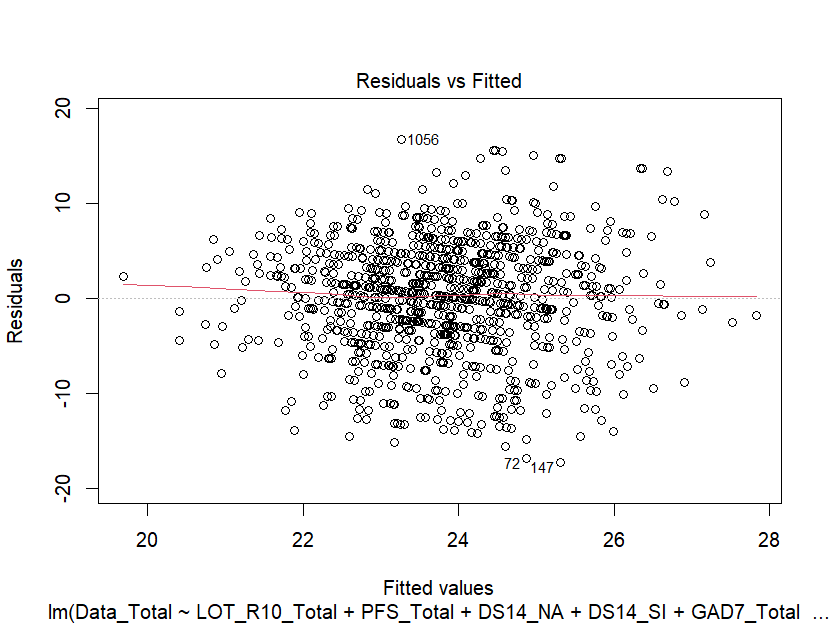

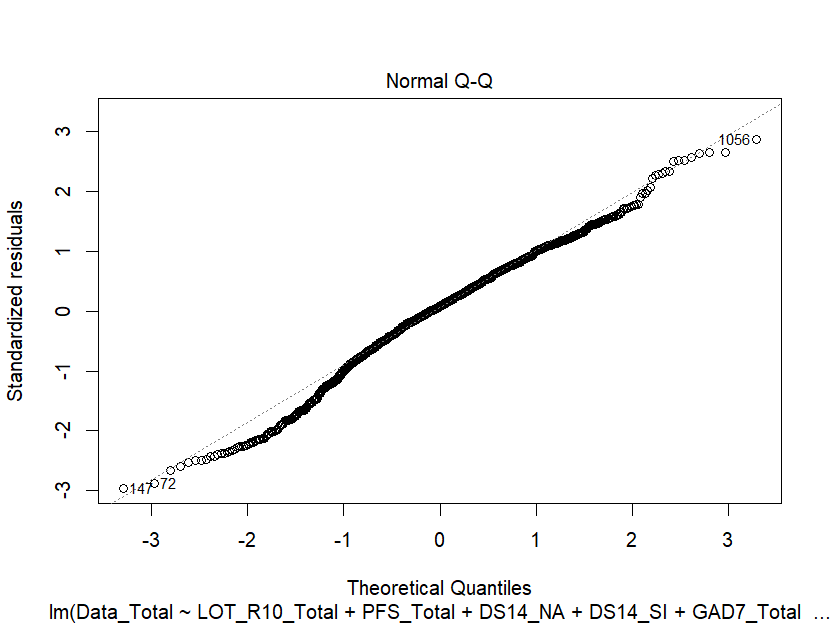
**

**
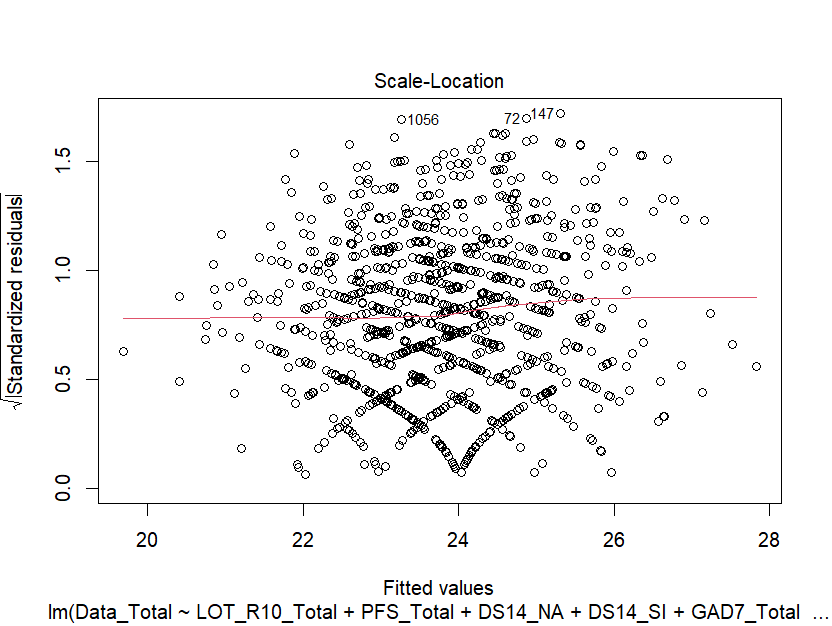

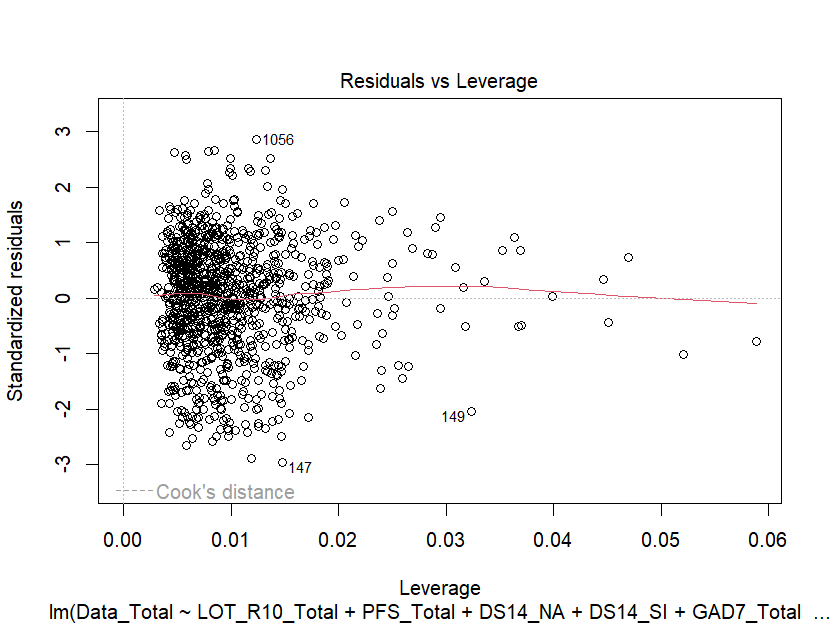
**

**Fig. S1.** Assumptions regression analysis


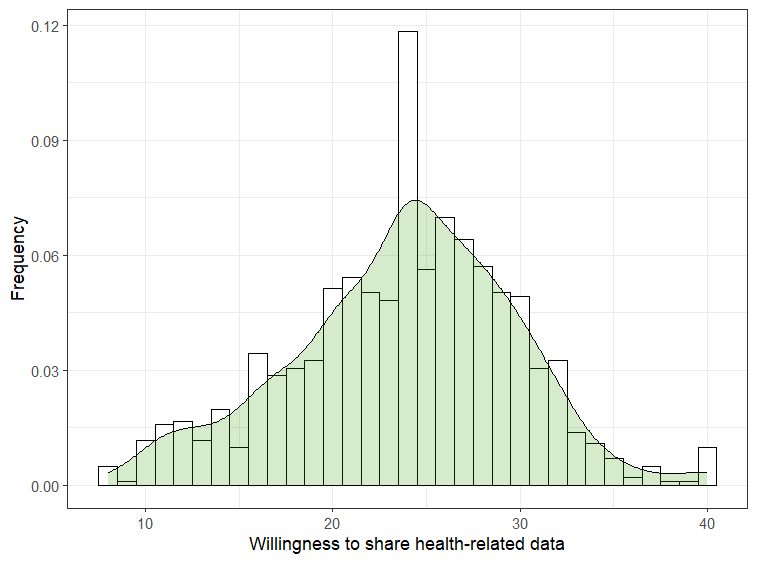


**Fig. S2.** Histogram and densityplot of willingness to share data

**
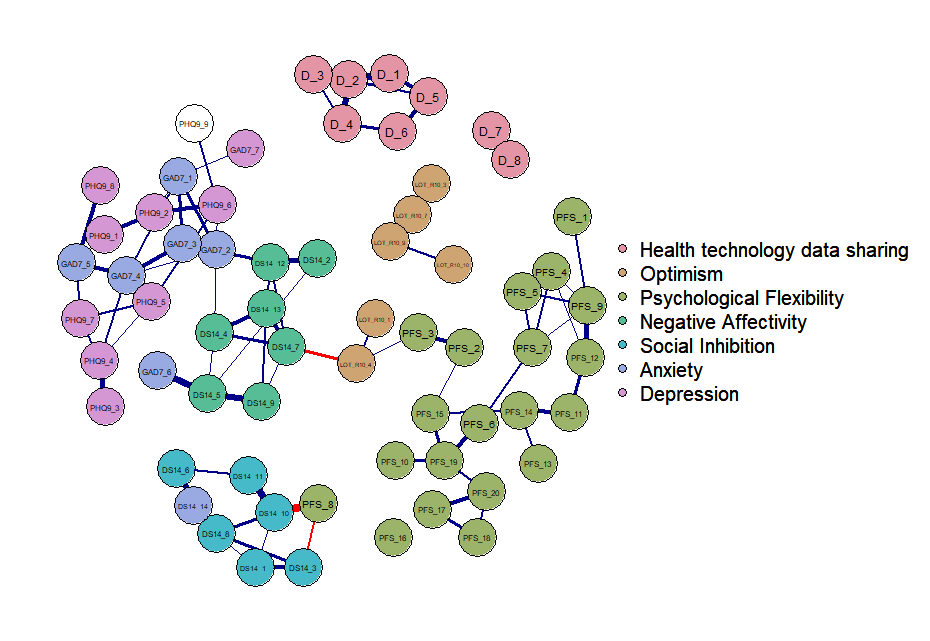
**

**Fig. S3.** Regularized correlation structure of all scales. It represents the relative importance of a node in the network, indicating the extent to which this node is related to other nodes in the network. Again, each construct is represented by a different colour, while each edge represents the relation between two items adjusted for the influence on all other items. In this network, there are no edges between the data sharing items and the psychological predictors, while within data sharing items 7 and 8 are again distinct from the other items within this domain. This indicates that the correlations between the items on optimism and psychological flexibility on the willingness to share health data are not strong enough and might be spurious. Therefore, the results presented in the network analysis outlined in Figure 1 should be interpreted with caution.
